# Piperine Reduces Neoplastic Progression in Cervical Cancer Cells by Downregulating the Cyclooxygenase 2 Pathway

**DOI:** 10.3390/ph16010103

**Published:** 2023-01-10

**Authors:** Luana Pereira Cardoso, Stefanie Oliveira de Sousa, Juliana Prado Gusson-Zanetoni, Laura Luciana de Melo Moreira Silva, Barbara Maria Frigieri, Tiago Henrique, Eloiza Helena Tajara, Sonia Maria Oliani, Flávia Cristina Rodrigues-Lisoni

**Affiliations:** 1 São Paulo State University (Unesp), Institute of Biosciences, Humanities and Exact Sciences (IBILCE), São José do Rio Preto, 15054-000, Brazil, Department of Biology Science; 2 Department of Molecular Biology, School of Medicine of Sao José do Rio Preto (FAMERP), Sao José do Rio Preto 15090-000, Brazil; 3 São Paulo State University (Unesp), School of Natural Sciences and Engineering, Ilha Solteira, 15385-000, Brazil, Department of Biology and Animal Science

**Keywords:** gynecological cancer, herbal medicines, alkaloid, piperine

## Abstract

Cervical cancer is the fourth-most common type of cancer in the world that causes death in women. It is mainly caused by persistent infection by human papillomavirus (HPV) that triggers a chronic inflammatory process. Therefore, the use of anti-inflammatory drugs is a potential treatment option. The effects of piperine, an amino alkaloid derived from *Piper nigrum*, are poorly understood in cervical cancer inflammation, making it a target of research. This work aimed to investigate the antitumor effect of piperine on cervical cancer and to determine whether this effect is modulated by the cyclooxygenase 2 (PTGS2) pathway using in vitro model of cervical cancer (HeLa, SiHa, CaSki), and non-tumoral (HaCaT) cell lines. The results showed that piperine reduces in vitro parameters associated with neoplastic evolution such as proliferation, viability and migration by cell cycle arrest in the G1/G0 and G2/M phases, with subsequent induction of apoptosis. This action was modulated by downregulation of cyclooxygenase 2 (PTGS2) pathway, which in turn regulates the secretion of cytokines and the expression of mitogen-activated protein kinases (MAPKs), metalloproteinases (MMPs), and their antagonists (TIMPs). These findings indicate the phytotherapeutic potential of piperine as complementary treatment in cervical cancer.

## 1. Introduction

Cervical cancer was ranked as the fourth-most frequently diagnosed cancer and the fourth-leading cause of cancer death in women worldwide in 2020. Globally, most cases (58.5%) and deaths (70.4%) occur in areas with low levels of human development [1]. This is because most cases of cervical cancer are caused by persistent infection with carcinogenic human papillomavirus (HPV) genotypes [1,2], with a worldwide prevalence of approximately 90% of cervical carcinomas [1].

Persistent infection by HPV 16 and 18, which are high-risk subtypes, accelerates the progression of cervical cancer, by blocking the main inhibitors of cell proliferation and enhancers of apoptosis [3,4]. This occurs through regulatory mechanisms in which oncoprotein E7 binds to members of the retinoblastoma protein (pRB) family (p105, p107, p130), promoting their degradation, while oncoprotein E6 binds to and inactivates p53 via a ubiquitin-dependent pathway, resulting in uncontrolled cell cycle progression and loss of DNA repair mechanisms, with consequent accumulation of mutations creating genomic instability [5].

In addition, inflammation is triggered in the presence of the virus; when the infection persists, the inflammation that previously contained the virus becomes chronic and is responsible for progression to a neoplasm [2,6]. Chronic inflammation is present in about 20% of human cancers and is the main inducer of malignancy by promoting metastasis and angiogenesis [7,8].

The cyclooxygenase-2-prostaglandin E2 receptor signal transducer (PTGS2-PGE2-PTGERs) pathway is believed to be the central pathway involved in chronic inflammation associated with oncological transformation in gynecological cancers. Data demonstrate an upregulation of this pathway in HPV-induced cervical cancer [8]. This regulation is mediated by the release of cytokines such IL-1β, IL-6, IL-8, and amphiregulin by the virus presence, which stimulates the protein kinase (MAPK) cascade, which in turn activates transcription factors AP-1 and NF-κB that are responsible for activating transcription of cyclooxygenase 2 (PTGS2) [9,10,11]. Cyclooxygenase 2 is an enzyme that catalyzes the synthesis of prostaglandin E2 (PGE2), and it functions by binding to its respective G protein-coupled membrane receptors, namely PTGER1, PTGER2, PTGER3, and PTGER4, each of which signals a response [12]. Receptors 2, 3, and 4 are most involved in cervical cancer [13,14,15,16,17]. Exacerbated activation of the PTGS2-PGE 2-PTGERs pathway triggers increased proliferation, cell survival, angiogenesis, and metastasis [7,18,19]. 

The metastasis process is the metastatic process is regulated by the action of matrix metalloproteinases (MMPs) [20]. These molecules are proteolytic proteins that degrade the extracellular matrix (ECM), contributing to metastasis, angiogenesis, tissue repair, and inflammatory processes. MMP2 and MMP9 are involved in the proteolytic degradation of components of the ECM, which allows for detachment of cells from the matrix and from the tissue itself, favoring their migration through the bloodstream to other tissues and organs. MMP activity is contained by MMP inhibitors (TIMPs) [21,22], and studies show that high levels of TIMP2 are associated with a better clinical outcome in patients with solid tumor [20]; therefore, the effect of treatment on this mediator has a substantial impact on controlling the progression of tumorigenesis.

Regarding the role of inflammation, the anti-inflammatory effect of the PTGS2 pathway is promising as a therapeutic alternative in cancer treatment [23]. Evidence shows that daily intake of acetylsalicylic acid (Aspirin) or other non-steroidal anti-inflammatory drugs (NSAIDs) reduces the risk of developing several types of cancer, including cervical cancer, at a reduction is 54% [24]. In addition, Aspirin/NSAIDs reduce angiogenesis and induce apoptosis [25]. The mechanism underlying the anti-inflammatory action of Aspirin is irreversible inhibition by acetylation of PTGS1 and PTGS2 enzymes [26].

Considering the efficacy of inflammation as a therapeutic target in cancer, other anti-inflammatory agents are being investigated, such as the alkaloid piperine. This alkaloid exhibits an inhibitory effect on PTGS2 and a higher affinity (−7.8 kcal mol^−1^) and energy binding (−85.08 kcal mol^−1^) than Aspirin and celecoxib [26]. Other data highlight the anti-inflammatory effects of piperine in suppressing the PTGS2 pathway in human osteoarthritis chondrocytes [27], brain ischemia-reperfusion-induced inflammation, and inflammation in human keratinocyte cells after UV-B irradiation [28]. However, inflammatory pathways involved in the action of piperine have not been extensively investigated in cases of neoplasms.

Piperine (1-piperoylpiperidine) is an amino alkaloid that is considered the main metabolite derived from the fruit of *Piper nigrum* (black pepper) and *Piper longum* (long pepper) [29]. In recent decades, phytomedicines have played a key role in drug discovery, where 50% of new drugs approved by the Food and Drug Administration (FDA) are of natural origin [30]

The antitumorigenic properties of piperine have been evidenced in different types of cancer, and it has been shown to target pathways involved in the cell cycle and apoptosis [29]; however, little is known about the role of piperine in cancer inflammation, especially in cervical cancer. Therefore, this is an important mechanism to investigate. The aim of this research was to investigate the in vitro antitumor effect of piperine on cervical cancer and to determine whether this effect is negatively modulated by cancer-associated inflammatory pathways

## 2. Results

### 2.1. Piperine Alters Morphology, Viability, and Cell Proliferation

**Figure 1:**
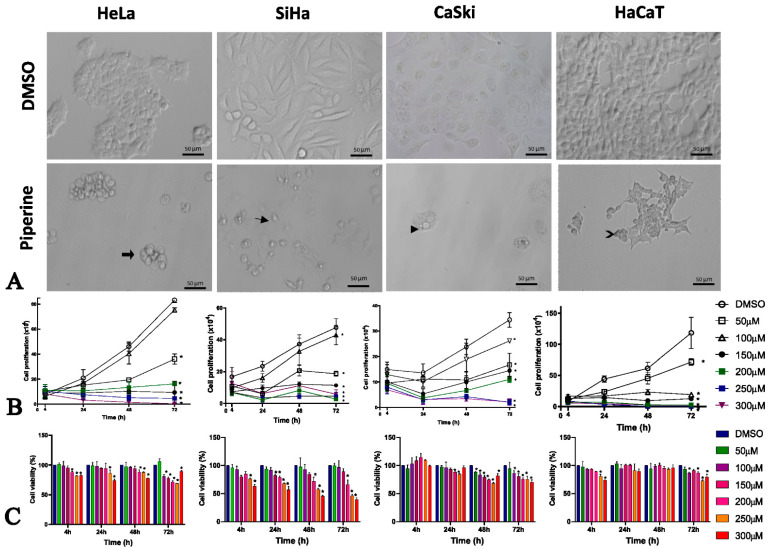
Piperine alters cell morphology, proliferation, and viability. (**A**) Photomicrograph of the morphology of HeLa, SiHa, CaSki, and HaCaT cell lines after 24 h of treatment compared with control (DMSO) and piperine [150 µM]. The thick arrow and chevron arrow point to blebs, the arrowhead indicates intracellular vesicles, and the thin arrow shows membrane disruption. (**B**) Representative graph of cell proliferation after treatment with piperine at concentrations of 50, 100, 150, 200, and 300 µM after, 4, 24, 48 and 72 h of treatment. (**C**) Viability of cells treated with piperine at concentrations of 25, 50, 100, 150, 200, 250, and 300 µM and after 24, 48, and 72 h of treatment. * vs. control, *p* < 0.05.

Our results revealed that piperine altered the morphology of all cell lines studied. In the HeLa and HaCaT lines, piperine reduced cell size, and the previously fusiform and stellate cells acquired a rounded shape with membrane vacuoles (blebs), as shown in the thick arrow and the chevron arrow in Figure 1A. In the SiHa cell line, the fusiform cells became rounded and amorphous due to removal of the cell contour, which was probably caused by rupture of the plasma membrane indicated by the small arrow. In the CaSki line, formation of intracellular vesicles (arrowhead in Figure 1A) was observed after treatment with piperine.

Cell proliferation was significantly reduced by piperine in high concentrations. As shown in Figure 1B, piperine reduced cell proliferation, starting at a concentration of 50 µM, in all cells except for HeLa cells, where a concentration of 100 µM piperine did not change cell proliferation.

Piperine reduced viability at high concentrations in tumorigenic cells from 24 h, but in the non-tumorigenic cells (HaCaT), piperine only had this effect at 72 h. This observation demonstrates that treatment durations of 24 h and 48 h would help to prevent the cytotoxic effect of piperine on non-tumor cells (Figure 1C).

The piperine IC_50_ varied according to the cell line studied, with values between 42.92 µM and 218.4 µM, as shown in Table 1. Studies have shown that the treatment doses in other tumor cell lines vary from 75 µM to 200 µM at incubation times of 24 h and 48 h [29], corroborating the IC_50_ in cervical cancer at these durations. Interestingly, in HaCaT, the IC_50_ at 24 h was the highest (218 µM), indicating that lower concentrations would not cause cytotoxicity. Therefore, due to the rapid bioavailability of piperine [31], the treatment duration of 24 h and the concentration of 150 µM were chosen because they produced an IC_50_ that was much lower than the IC_50_ of non-tumor cells and represented the bioactive concentration in tumor cells.

### 2.2. Piperine Inhibits Colony Formation, Impairs Cell Cycle Progression, and Induces Apoptosis by DNA Fragmentation

**Figure 2:**
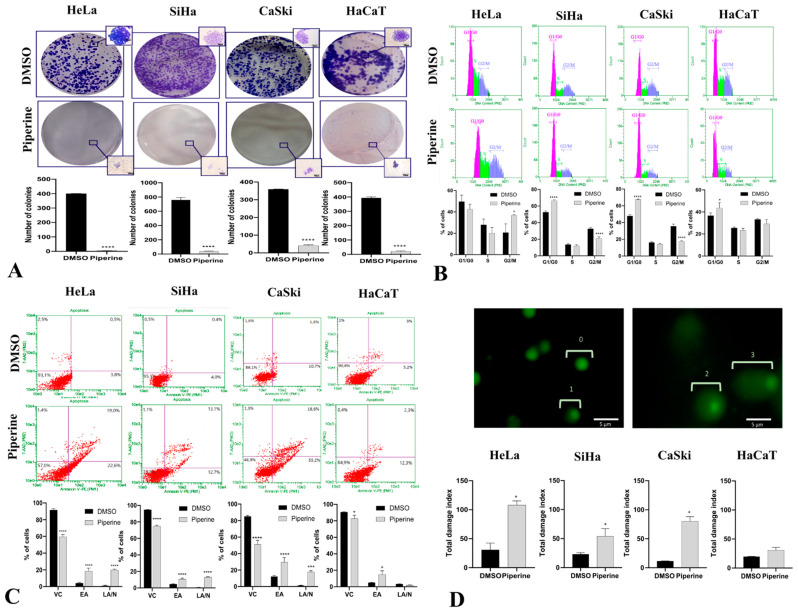
Piperine inhibits colony formation, impairs cell cycle progression, and induces apoptosis by DNA fragmentation. (**A**) Photograph of colony formation and number of cells per colony in SiHa, HeLa, CaSki, and HaCaT lines after 14 days of treatment compared with control (DMSO) and with piperine [150 µM]. (**B**) Cell cycle analysis in SiHa, HeLa, CaSki, and HaCaT lines after treatment with control (DMSO) and piperine [150 µM]. (**C**) Representative plots of apoptotic cell death assessed by annexin V-PE and 7-AAD fluorescence in flow cytometry, where annexin V-PE (-) and 7-AAD (-) represents viable cells (VCs), annexin V-PE (+) and 7-AAD (-) represents early apoptosis (EA), and annexin V-PE (+) and 7-AAD (+) represents late apoptosis (LA) or necrosis (N). (**D**) Fluorescence photomicrographs of CaSki cell nuclei with different types of damage assessed by the comet assay: 0 = no apparent damage; 1 = damage 1, with one times the nucleus size; (2) damage 2, with two times the size of the core; and (3) damage 3, with two or more times the size of the core. The graphs represent the damage index in SiHa, HeLa, CaSki, and HaCaT cells after treatment with control (DMSO) and piperine [150 µM], * vs. control *p* < 0.05, *** vs. control, *p* < 0.001, **** vs. control, *p* < 0.0001.

Piperine suppressed cell proliferation in HeLa, SiHa, CaSki, and HaCaT lines, preventing the formation of colonies (Figure 2A). Piperine regulated this process by stopping the cells at cell cycle checkpoints; in CaSki, SiHa, and HaCaT cells, this occurred in the G1/G0 phase (Figure 2B), and in HeLa cells, piperine led to cell arrest in the G2/M phase, which differs from the others because it is secretory glandular epithelial cell line.

During arrest in the G1/G0 and G2/M phases, cells undergo molecular processes to determine the most favorable fate: self-renewal, differentiation, or death [32,33]. In this case, the cells were destined to die through a programmed cell death mechanism called apoptosis. The results indicate that piperine caused apoptotic programmed cell death in the four investigated cell lines (Figure 2C); however, in non-tumor cells, piperine did not cause late apoptosis as it had less detrimental effects. DNA fragmentation is a process present in apoptosis that was detected via the comet assay, in which piperine treatment caused DNA fragmentation in tumor cells but was not genotoxic to non-tumor cells (Figure 2D).

### 2.3. Piperine Reduces Cell Migration in Cervical Cancer through Regulation of Matrix Metalloproteinases and Their Antagonists

**Figure 3:**
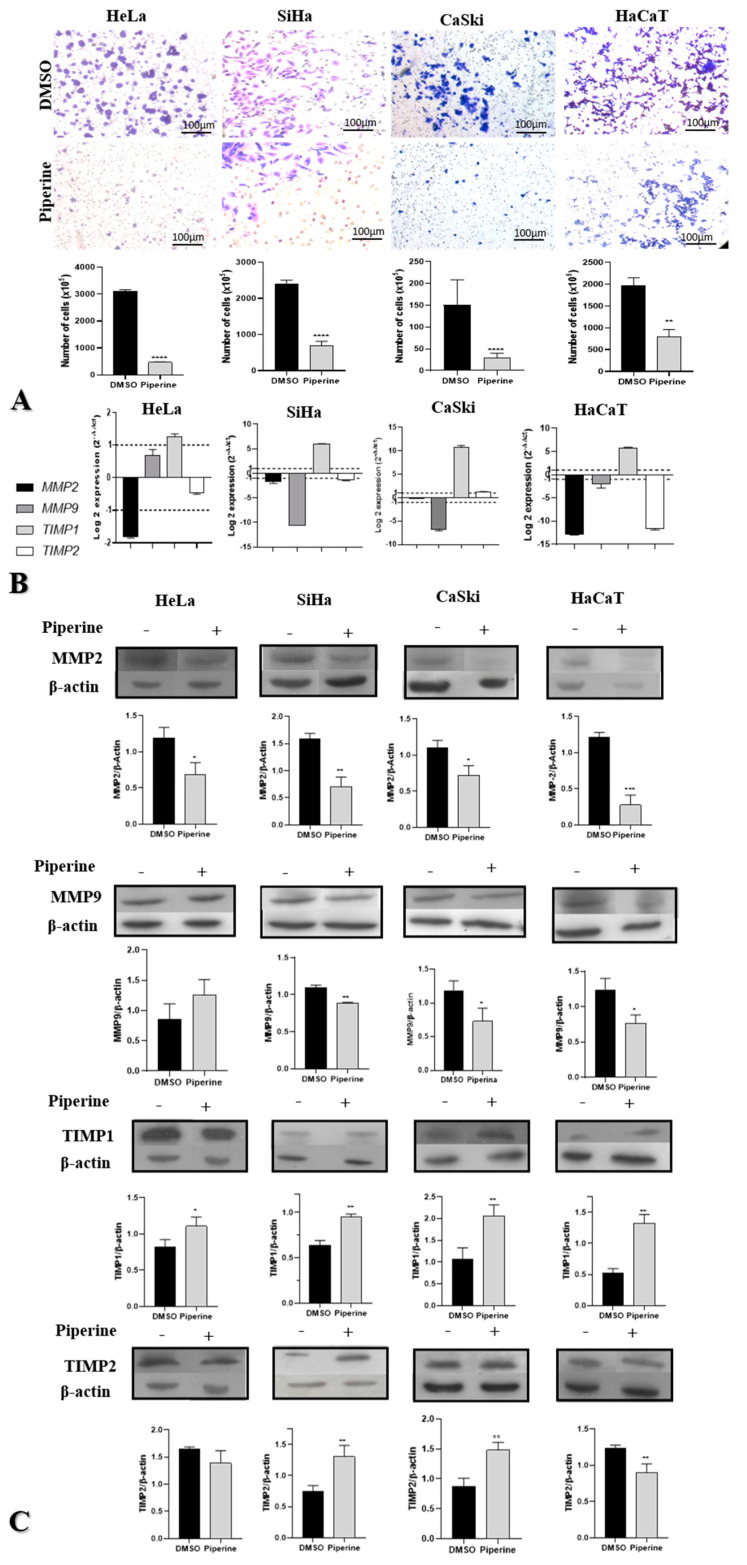
Piperine reduces cell migration in cervical cancer through the regulation of matrix metalloproteinases (MMPs) and their antagonists. (**A**) Photomicrograph of the migration of HeLa, SiHa, CaSki, and HaCaT cells in the lower chamber of the transwell after 24 h of treatment with control (DMSO) and piperine [150 µM]. The respective statistical graphs are shown. (**B**) Graphs of MMP2, MMP9, TIMP1, and TIMP2 mRNA expression after piperine treatment compared with control in HeLa SiHa, CaSki, and HaCaT cells. The dotted line (≥1.0 or ≤-1.0) is equivalent to the significant expression difference based on log 2. (**C**) Graphs of MMP2, MMP9, TIMP1, and TIMP2 protein expression evaluated by Western Blot in HeLa SiHa, CaSki, and HaCaT cells after treatment with control (DMSO) and piperine [150 µM]. * vs. control, *p* < 0.05, ** vs. control, *p* < 0.01, *** vs. control, *p* < 0.001, **** vs. control, *p* < 0.0001.

In the three tumor cells (HeLa, SiHa, and CaSki), piperine significantly reduced cell migration; in contrast, in the non-tumorigenic lines (HaCaT), this reduction was less intense (Figure 3A). Therefore, the phytotherapeutic effect of piperine could be selective.

Piperine has been shown to regulate MMP2, MMP9, TIMP1, and TIMP2 in cervical cancer cells at the gene and protein levels. The MMP9 gene and its respective protein were downregulated in SiHa, CaSki, and HaCaT cells. In HeLa, SiHa, and HaCaT cells, piperine caused a reduction in the expression of MMP2 messenger RNA, whereas the protein encoded by this RNA was downregulated by piperine in all the analyzed cell lines. At the gene and protein levels, TIMP1 was overexpressed in the four cell lines following treatment with piperine. In the case of TIMP2, piperine regulated gene expression positively in CaSki cells and negatively in non-tumor cells (HaCaT); regarding protein expression, there was an increase in expression in CaSki and SiHa cells and a decrease in expression in HaCaT cells after treatment with piperine (Figure 3B,C).

### 2.4. Piperine Reduces Tumorigenesis by Regulating the PTGS2 Inflammatory Pathway and Cytokine Secretion

**Figure 4:**
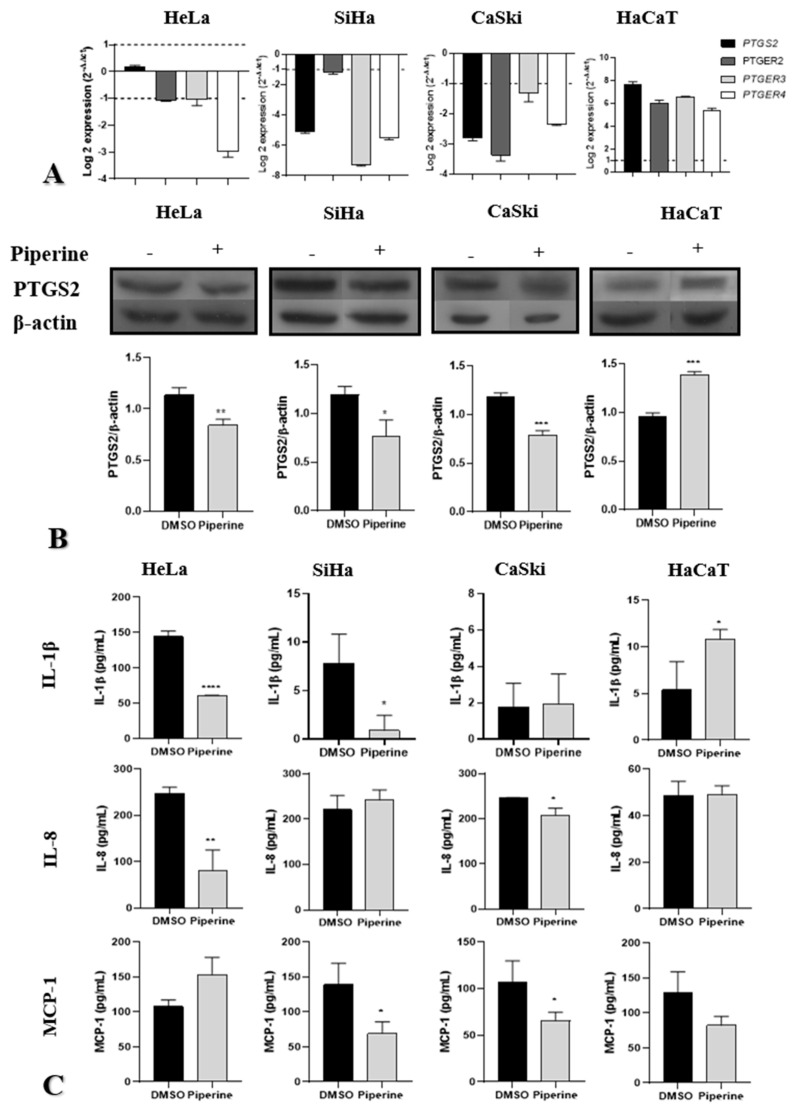
Piperine reduces tumorigenesis by regulating the PTGS2 inflammatory pathway and cytokine secretion. (**A**) Graphs of PTGS2, PTGER2, PTGER3, and PTGER4 mRNA expression after piperine treatment compared with the control in HeLa SiHa, CaSki, and HaCaT cells. The dotted line (≥1.0 or ≤−1.0) is equivalent to the significant expression difference based on log 2. (**B**) Graphs of PTGS2 protein expression evaluated by Western Blot in HeLa SiHa, CaSki, and HaCaT cells after treatment with control (DMSO) and piperine [150 µM]. (**C**) Graphs of the colorimetric ELISA assay for analysis of cytokines/chemokines IL-1β, IL-8, and MCP-1 secreted by SiHa, HeLa, CaSki, and HaCaT cells after treatment with control (DMSO) and piperine (150 µM). * vs. control, *p* < 0.05, ** vs. control, *p* < 0.01, *** vs. control, *p* < 0.001 **** vs. control, *p* < 0.0001.

Piperine downregulated PTGS2 gene and protein expression in CaSki and SiHa cells and downregulated only protein expression in the HeLa lineage. The receptors PTGER2, PTGER3, and PTGER4 exhibited reduced gene expression in all tumor cells, indicating a reduction in the action of PGE2 on these receptors (Figure 4A,B). In non-tumor cells (HaCaT), the effect was inverse, with an upregulation of PTGS2 and its receptors (PTGER2, PTGER3, and PTGR4) (Figure 4A,B), indicating the pro-inflammatory effect of piperine on non-tumor cells.

Regarding cytokine secretion, piperine reduced IL-1β secretion in HeLa and SiHa cells, did not produce significant changes compared with the control in CaSki cells, and increased secretion in non-tumor cells (HaCaT). IL-8 secretion was reduced in HeLa and CaSki cells after treatment with piperine; however, there was no change in secretion in SiHa and HaCaT cells. Piperine reduced MCP-1 chemokine levels in SiHa and CaSki cells but did not alter levels HaCaT and HeLa cells (Figure 4C).

### 2.5. Piperine Reduces the Expression of HPV16 Oncogenes in CaSki Cells

**Figure 5:**
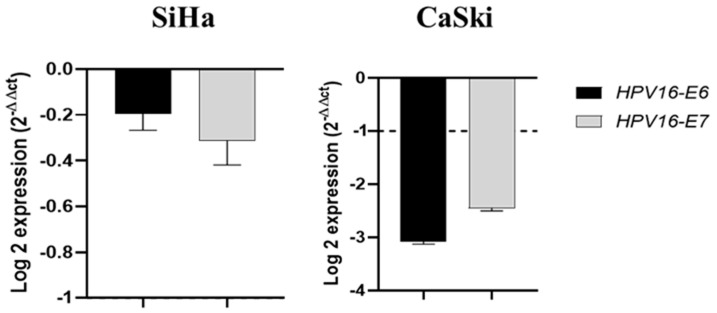
Piperine reduces the expression of HPV16 oncogenes in CaSki cells. Graphs of HPV16-E6 and HPV16-E7 mRNA expression after piperine treatment compared with the control in SiHa and CaSki cells. The dotted line (≥1.0 or ≤−1.0) is equivalent to the difference of the significant expression based on logarithm 2.

As shown in Figure 5, piperine significantly reduced the expression (four- to six-fold) of E6 and E7 of HPV16 oncogenes in CaSki cells, but did alter expression levels in SiHa cells. HPV16 oncogenes were not evaluated in HeLa cells because they are infected by HPV18 and not 16, while HaCaT cells are not infected by any of these viruses.

### 2.6. Piperine Modulates the Expression of p38 and ERK (MAPKs)

**Figure 6:**
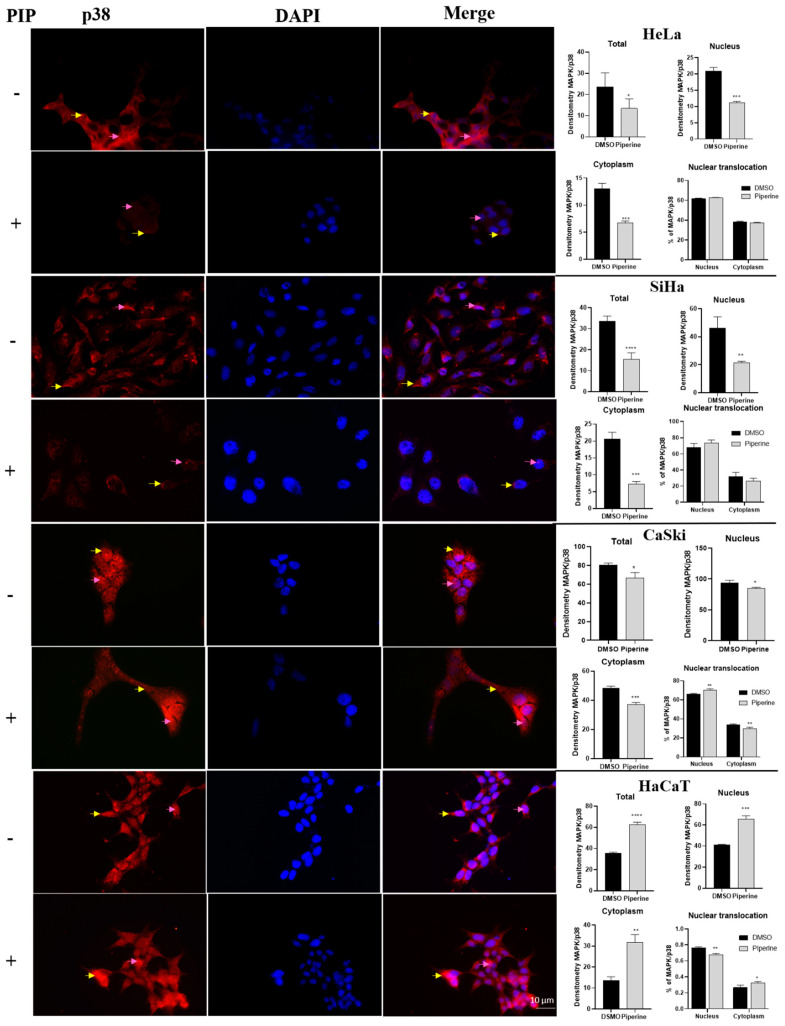
Immunocytochemistry of p38/MAPK protein expression is indicated by a yellow arrow in the cytoplasm and pink in the nucleus. Graphs show the densitometry in each cell and nuclear translocation compartment after treatment with control (DMSO) and piperine [150 µM]. * vs. control, *p* < 0.05, ** vs. control, *p* < 0.01, *** vs. control, *p* < 0.001, **** vs. control, *p* < 0.0001.

There was a reduction in p38 expression in tumor cells (HeLa, SiHa, and CaSki) in all cell compartments analyzed (entire cells, nucleus, and cytoplasm). Furthermore, piperine increased p38 nuclear translocation exclusively in CaSki cells (Figure 6). In contrast, in non-tumor cells (HaCaT), p38 expression significantly increased both in the nucleus and in the cytoplasm. Although piperine increased p38 expression in these cells, it reduced nuclear translocation, resulting in a lower percentage of active p38 in the nucleus than in the cytoplasm.

**Figure 7:**
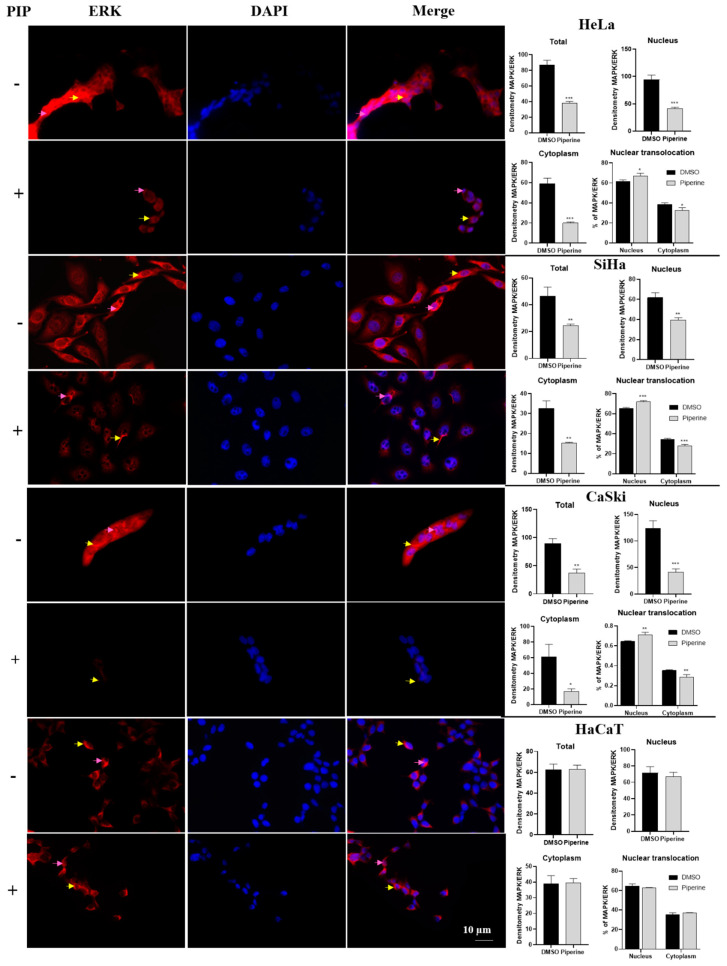
Immunocytochemistry of ERK/MAPK protein expression is indicated by a yellow arrow in the cytoplasm and pink arrow in the nucleus. Graphs showing densitometry in each cell and nuclear translocation compartment after treatment with control (DMSO) and piperine [150 µM]. * vs. control, *p* < 0.05, ** vs. control, *p* < 0.01, *** vs. control, *p* < 0.001.

ERK expression was reduced in all cell compartments of cervical cancer cell lines (Figure 7). However, although piperine reduced ERK expression, there was an increase in the nuclear translocation of this protein after piperine treatment. In contrast, in HaCaT cells, ERK expression, and translocation were not altered by the action of piperine, justifying the less pronounced effects on the cell cycle and proliferation of these cells.

## 3. Discussion

Increased cell proliferation constitutes the main mechanism that triggers carcinogenesis and represents an important target of cancer therapies [34]. The results demonstrated the effects of piperine on cell proliferation, which was significantly reduced at different concentrations of piperine on the growth curve and colony formation. The mechanism triggered by piperine that reduced this property was the arrest of cells in different phases of the cell cycle: epidermoid cells were arrested in the G1/G0 phase, and adenocarcinoma cells were arrested in the G2/M phase.

In molecular terms, this mechanism was regulated by the ERK protein, the expression of which was negatively modulated by piperine. The ERK pathway, when upregulated, increases cell proliferation by activating transcription factors, such as cAMP response element binding protein (CREB), Myc-like transcriptional regulator (c-Myc), AP-1, and NF-κB, which act on genes that induce cell proliferation [35,36]. 

Other molecular mediators associated with cell proliferation are the cytokines IL-1β and IL-8, which are excessively secreted in cervical cancer and contribute to metastasis, angiogenesis, and cell survival [37,38,39,40,41,42]. Furthermore, HPV16 E6 and E7 oncogenes are associated with these mechanisms as they degrade mediators involved in the control of cell proliferation and apoptosis [5]. Therefore, the decreased expression of these cytokines and of E6 and E7 by the action of piperine was responsible for the reduction in these cellular events.

Control of cell survival is a property that maintains the physiological homeostasis of cells; when a cell is damaged or genetically altered without possible repair, it undergoes programmed cell death. In cancer, there is a lack of control of this mechanism, which allows for the survival of mutated cells for tumor progression [33,43]. Our results indicate that piperine contained cell viability at different concentrations and that the associated death mechanism was apoptotic programmed cell death.

Apoptosis is characterized by molecular events that cause cell shrinkage, loss of organelles, formation of membrane vacuoles (blebs), and DNA fragmentation [44]. These morphological characteristics are well defined and were evident in our morphology assay. DNA fragmentation was demonstrated by the comet assay, where the comet tail is proportional to the amount of fragmented DNA [44]. The apoptotic and genotoxic results showed less intense effects of piperine in HaCaT cells, in which there was no late apoptosis, necrosis, or genotoxic effect on the DNA. This result is positive, as late apoptosis and necrosis cause toxicity to normal cells during chemotherapy treatments [45]. 

Similar results about apoptosis and cell cycle were obtained in other cell lines. In colon, breast, rectal, and melanoma cancers, piperine reduced cell proliferation and viability from the arrest of cells in the G1/G0 phase of the cell cycle, with consequent induction of apoptosis [46,47,48,49,50,51,52]. In human leukemia cells, the same process was reported; however, the cells accumulated in the S phase of the cell cycle [53]. In oral cancer, ovarian cancer, and osteosarcoma cells were arrested in the G2/M phase [54,55].

Metastasis is a cellular biological process with several steps involving cascades that induce migration and invasion, allowing the spread of cancer cells to other tissues and organs [56,57]. When cancer progresses to metastasis, it significantly reduces the survival of patients, and about 90% die [58]. According to our results, piperine reduced cell migration by regulating MMP2 and MMP9 and their action on their respective antagonists, as TIMP1 and TIMP2 inactivate metalloproteinases and prevent their action, triggering a reduction in tumor cell migration [21]. Overexpression of MMP2 and MMP9 was observed in tissue samples and cervical plasma levels and has been shown to be related to poor prognosis in the disease [20,59,60]. Therefore, the action of piperine on these MMPs represents an excellent response to controlled cell migration.

When excessively secreted, the inflammatory cytokines IL-1β, IL-8, and MCP-1 signal the synthesis of metalloproteinases and subsequently increase migration [37,38,39,42,61,62] Therefore, while piperine negatively modulated MMPs, it also reduced the secretion of these cytokines, exerting a dual response over metastatic control.

In other studies, the same effect was demonstrated, where piperine controlled the metastatic reduction by regulating MMPs and TIMPs. In osteosarcoma, piperine inhibited cell migration by reducing the expression of MMP2 and MMP9 and increasing the expression of TIMP1 and TIMP2 [55]. Similarly, in triple-negative breast cancer, piperine inhibited cell migration by downregulating the expression of MMP2 and MMP9 [63]. In metastatic prostate cancer, piperine regulated the Akt/mTOR/MMP-9 signaling pathway and reduced metastasis [64]. 

Inflammation is closely related to neoplastic genesis and progression, especially in cervical cancer, which is mostly caused by persistent HPV infection. Expression of the PTGS2 enzyme, its PGE2 product, and its receptors triggers the inflammatory response, which results in increased cell proliferation, cell survival, and metastasis [7,18,19]. These processes were contained by the action of piperine in our study due to its downregulation of the expression of PTGS2 and its respective receptors, thereby preventing the binding of prostaglandin and subsequent transduction of signals that lead to the occurrence of these events.

The PTGS2 pathway regulates and is regulated by the mediators investigated in this study. Other studies have shown that PTGS2 and PTGER4, when overexpressed, induce the expression of MMP2 and MMP9 and consequently enhance cell migration [65,66]. In our study, both mediators were expressed negatively, and migration was reduced. The PGE2 produced by PTGS2 regulates the expression of IL-1β, IL-8, and MCP-1 [38,67], and its regulation is mediated by these cytokines via positive feedback through MAPKs, ERK, and p38 as intermediaries [66,68,69,70]. All these mediators were downregulated by piperine in cervical tumor cells, indicating the anti-inflammatory effect of piperine via PTGS2.

**Figure 8:**
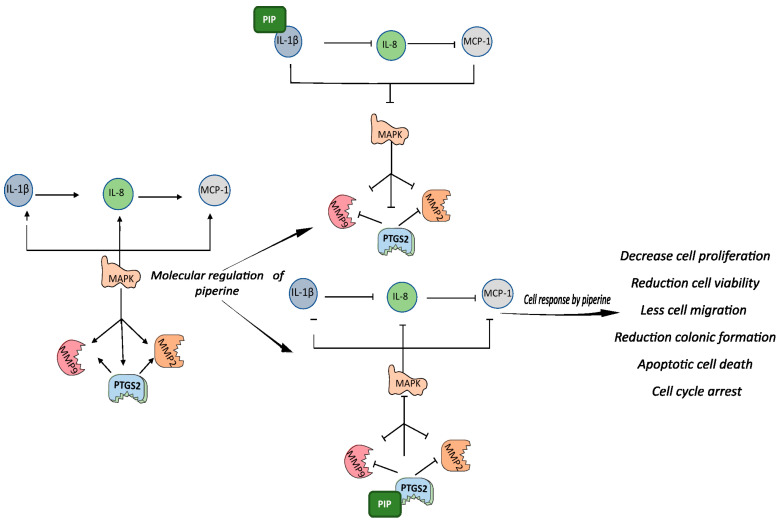
Graphic representation of piperine-mediated modulation of IL-1β and PTGS. Graphical abstract representation of the relationship between the studied pathways and the results: IL-1β cytokines induce the expression of IL-8, which in turn stimulates the expression of MCP-1. All these cytokines are regulated by PTGS2, which jointly regulates them via positive feedback, using the MAPK pathway as an intermediary. MMPs are also stimulated by cytokines and PTGS2. When piperine binds to PGS2, all those involved in the pathway are downregulated, and the same occurs when piperine binds to IL-1β. The molecular responses to piperine modulation result in reduced viability, proliferation, migration, and colony formation in addition to activation of apoptosis, DNA fragmentation, and cell arrest in the cell cycle.

Piperine may have regulated the PTGS2 pathway by binding to this protein. Molecular docking studies have shown direct binding of piperine to PTGS2 with a score of 5042 kcal/mol [71]. However, piperine also has a high binding affinity for IL-1β, which has a binding site for piperine with an affinity constant of 14.3 × 104 M −1a 298 K, and spontaneous interaction between these molecules has been observed (∆G = −25 kJ/mol) [72]. This evidence, together with the results of our study, supports the hypothesis that piperine may have bound to these mediators, preventing the binding of IL-1β to its receptor and the synthesis of PGE2, and resulting in an anti-inflammatory and antitumor effect in the tumor cells (Figure 8).

However, unlike our observations in tumor cells, the expression of IL-1β, PTGS2, and their receptors was positively regulated by piperine in HaCaT cells, indicating the pro-inflammatory action of non-tumor cells against this molecule, which can be considered a stress stimulus. This occurred because p38 is primarily responsible for the stress response, and it was upregulated by piperine, resulting in increased expression of IL-1β and PTGS2 that responded to this stimulus from inflammation [73]. This observation may explain the less intense effect of piperine on apoptosis, cell cycle, and cell migration observed in our study.

Inflammation is considered a contradictory process in cancer; when inflammation is chronic, the response worsens tumor progression, while in healthy cells, inflammation is a positive response aimed at eliminating a foreign agent or stressor [74]. In the latter case, considering the whole organism, inflammation in non-tumor cells could be a way to reduce the cytotoxic effects of piperine.

Regarding the anti-inflammatory effect of piperine in the literature, it was reported that this alkaloid reduced the expression of PTGS2, PGE2, and IL-1β in osteoarthritis models [27], reduced inflammation after UV-B irradiation [28], and reduced inflammation induced by cerebral ischemia-reperfusion [75]. In gastric epithelial cells infected by Helicobacter pylori, piperine impaired gene expression and IL-8 secretion [76]. In gastric cancer, piperine repressed IL-1β expression, resulting in inhibition of p38/MAPK and STAT3 activation [26]. In a mouse model of inflammatory bowel disease pre-treated with piperine, levels of MCP-1 and IL-1β were decreased [77].

These studies highlight the anti-inflammatory role of piperine on these gene pathways; however, there is a gap in the literature on the role of piperine in cancer, and this work has contributed to the understanding of the mechanisms of action of this alkaloid in cervical cancer inflammation. In this study, we observed a modulating effect on neoplastic progression by piperine acting via PTGS2 and IL-1β, which resulted in negative modulation of p38, ERK, other cytokines (IL-8 and MCP-1), MMP9, and MMP2. These mediators, once negatively expressed, triggered decreased viability, migration, cell proliferation, colony formation, and increased cell cycle arrest and apoptosis.

Our work has some limitations, one of which is the lack of a non-tumorigenic cell line of the uterine cervix, for being a more adequate comparison control. Another limitation is the lack of protein expression analysis of PGE2 receptors that could be studied in future work. Our study demonstrates very promising results regarding the mechanism underlying the action of piperine, but it is not known whether the effects will be reproduced at the organism level. Therefore, future studies analyzing the differentially expressed mediators in an animal model will be very welcome and will help in the future application of piperine in cancer therapies.

## 4. Materials and Methods

### 4.1. Materials

Cell lines: three tumorigenic strains and one non-tumorigenic strain were used. The strains are from the American Tissue Cell Culture (ATCC): squamous cell carcinoma of the cervix (SiHa), adenocarcinoma of the cervix (HeLa) and squamous cell carcinoma of the cervix (CaSKi). The tumorigenic lineage studied was the keratinocyte cell line derived from human skin (HaCaT).

Piperine: the compound piperine (Sigma-Aldrich, St. Louis, MO, USA) was used at a concentration of 25, 50, 100, 150, 200, 250, and 300 μM, diluted in DMSO at concentrations below 0.85% in the tests of cell viability and proliferation and in subsequent assays at concentrations below 0.5%, concentrations that are not toxic to the cells studied [78]. 

### 4.2. Treatment

For all experiments, the cells were cultured in culture dishes at appropriate cell concentrations for each experiment, in the plating the complete medium with 10% fetal bovine serum (FBS) was used. After 24 h, the medium was replaced with serum-free medium in order to synchronize the cells, and after 24 h, the treatment was inserted into the cells with complete medium. In the initial assays, piperine was applied at concentrations of 25, 50, 100, 150, 200, 250, and 300 μM, DMSO was used as a negative control and the treatment times in the cells were 4, 24, 48, and 72 h. From the cell migration assay, a time and concentration of treatment with better performance and selectivity to tumor cells was chosen. The cells of the experiments were kept in an oven at 37 °C and an atmosphere of 5% CO_2_. All assays were performed in three independent experiments and in triplicates and data were statistically analyzed by the GraphPad Prism 8.0.1.

### 4.3. Cultivation and Analysis of Cell Morphology

HeLa and SiHa cell lines were seeded in MEM (E), CaSKi in RPMI 1640 and HaCaT in DMEM, all supplemented with 10% bovine serum, 10 mM non-essential amino acids, 100 mM sodium pyruvate and 1mM antibiotic/antimycotic. Cell growth and morphology were evaluated daily under an inverted microscope and photographed, when cell density was high (between 70 and 90%), the material was trypsinized and subdivided into replicates.

### 4.4. Cell Proliferation

Cells (5 *×* 10^4^) were grown in 24-well culture plates, seeded in 300 µL of complete medium. Cells were trypsinized, stained with Trypan Blue, and counted in the Countess Automated Cell Counter II (Life Technologies) cell counter. To analyze the proliferation index, a growth curve was performed for each concentration to be tested after 4, 24, 48, and 72 h of treatment. Afterwards, the growth curves were statistically analyzed by two-way analysis of variance (ANOVA) and Dunnett’s test (GraphPad Prism 8.0.1.), with *p* < 0.05 considered a significant difference.

### 4.5. Cytotoxicity and Cell Viability Assay

The assay was performed using the MTS reagent—CellTiter 96 AQueous One Solution Cell Proliferation Assay (PROMEGA, Madison, WI, USA), according to the manufacturer’s standards. Cells (5 × 10^3^) were plated in 96-well plates containing 100 μL of complete medium. After the treatment times, the cells received 20 μL of the MTS solution and after 3 h of reaction, the reading was performed in a spectrophotometer at a wavelength of 490 nm (Thermo Plate, Tp-Reader Basic).

To determine the IC_50_ (50% Inhibitory Concentration of Cells), using the function in the GraphPad Prism 8.0.1 application. For cell viability analysis, two-way analysis of variance (ANOVA) and Dunnett’s test (GraphPad Prism 8.0.1.), with *p* < 0.05 considered a significant difference.

### 4.6. Cell Migration Assay

Cells (5 × 10^4^) were added to the upper compartment of transwell inserts with 8 µm diameter pore membrane (BD—Biosciences San Jose, CA, USA) containing 200 µL of serum-free medium and treatments. In the lower compartment, 750 µL of complete medium with fetal bovine serum was added. AfterwardsAfterward, the cells were incubated in an oven for 24 h for migration to occur, followed by fixation in paraformaldehyde and staining in violet crystal. The inserts were analyzed and photographed in five different fields. The results were analyzed using the t test, with *p* < 0.05 considered a significant difference.

### 4.7. Colony Formation Assay in Liquid Medium

Cells (8 *×* 10^2^) were grown in six-well plates containing complete medium. After 12 h of culture, the treatment was added and the medium and treatment were replaced every two days. After 14 days of culture, the cells were fixed in methanol and stained with crystal violet. Colonies were photographed and counted by visual inspection and the number of cells per colonies colony, but by microscopy. Finally, the data were subjected to statistical analysis by the t test t-test, with *p* < 0.05 considered a significant difference.

### 4.8. Alkaline Comet Assay

Cell pellets (5 × 10^4^) were mixed with low melting agarose low melting point and added onto slides containing normal melting point agarose. The lysis step was carried out to rupture the membranes and sequentially a run-in electrophoresis for 20 min at 25 V and 300 Kva in alkaline buffer solution. Finally, the cells were neutralized and fixed in 100% ethyl alcohol. The slides were stained with a 1× Gel Red solution, and analyzed under a fluorescence microscope at 400× magnification. An amount of 100 cells were analyzed per experimental group. The cellular nuclei were classified in a damage class that is determined according to the intensity and size of the comet tail and submitted to a formula to determine the damage index.

### 4.9. Apoptosis and Cell Cycle Analysis

Cells were analyzed by flow cytometry (Guava Easy Cyte, MILLIPORE). For the analysis of Apoptosis and Cell Necrosis, cell suspensions (1 *×* 10^6^) were incubated with monoclonal antibody ANXA5 conjugated to fluorochrome PE (BD Phar-migen, San Diego, CA, USA) and with 7-ADD following the manufacturer’s protocol. For Cell Cycle analysis, cells (1 × 10^6^) were fixed with 70% ethanol and resuspended in Guava^®^ Cell Cycle Reagent (MILLIPORE, USA) following the manufacturer’s protocol. Then the cells were analyzed by flow cytometry (Guava Easy Cyte, MILLIPORE) and the data submitted to two-way analysis of variance (ANOVA) and the Sidak test.

### 4.10. RNA Extraction and Quantification

RNA from control and piperine-treated cells was extracted with the RNeasy^®^ Mini Kit (QIAGEN Group, USA) following the manufacturer’s protocol and quantified in a Nanodrop ND-1000 spectrophotometer (Thermo Scientific, Wilmington, DE, USA) and evaluated for to integrity by the presence of the two ribosomal bands 18S and 28S in a 1% agarose gel. The cDNA (complementary DNA) was obtained by reverse transcription reaction with the High Capacity cDNA Reverse Transcription Kit system (Applied Biosystems, Forster City, CA, USA) following the manufacturer’s protocol.

The genes studied in this project are evident in Table 2 with their respective sequences. The genes were previously selected for being related to the cervical tumor process [79] (HEMMAT; BAGHI, 2018), together with our hypothesis of the anti-inflammatory action of piperine mediated by this genetic pathway [27].

### 4.11. Real-Time PCR Analysis

The reactions were performed in a 7500 Fast Real-Time PCR System thermocycler (Applied Biosystems), at the Laboratory of Molecular Markers Biomarkers and Medical Bioinformatics, at the Faculty of Medicine of São José do Rio Preto, FAMERP, SP. All reactions were prepared in triplicate, including the endogenous control glyceraldehyde-3-phosphate dehydrogenase (GAPDH), which was used as normalizers, and processed in a final volume of 20 µL containing 200 ng of cDNA, SYBR^®^ Green PCR Master Mix and 100 nM of each primer, according to the Applied Biosystems protocol. Finally, the values of gene expression obtained in the analyzes were normalized by the result of the quantification of the control sample, chosen as a calibrator for all samples. The method 2^(−ddCt) of Livak (2001) [80] was used for the comparative analysis calculations.

### 4.12. Protein Extraction and Quantification

Proteins were extracted using RIPA pH 7.4 lysis buffer (Sigma cod R0278), protease inhibitor (Sigma cod P8340) and Na_3_VO_4_ phosphatase inhibitor (Sigma cod 450243), following the manufacturer’s protocol. Aliquots of these samples were subjected to quantification using the Pierce™ BCA Protein Assay Kit (Thermo Scientific, Wilmington, DE, USA).

### 4.13. Western Blotting

The lysed sample was diluted and normalized in H_2_O to obtain 30µg of proteins, β-mercapto (Sigma cod M6250), and 10% SDS added together with the samples. This solution was boiled in a dry bath at 100°C for 5 min. Proteins (30 µg) were electrophoretically separated on a 12% polyacrylamide gel, according to the MiniPROTEAN Tetra Cell protocol (Bio-Rad, Hercules, USA), and transferred to nitrocellulose membranes (Bio-Rad, Hercules, USA). The membranes were blocked with 5% powdered milk diluted in TBS-T (Bio-Rad Reagent, Hercules, USA), and kept for 1 to 12 h at room temperature under agitation. Then, the membranes were incubated overnight with the antibodies PTGS2 (1:500 μL Abcam, Cambridge, UK), MMP2 (1:500 μL, AB-clonal, Woburn, MA, USA), MMP9 (1:1000 μL, AB- clonal, Woburn, MA, USA), TIMP1 and TIMP2 (1:500 μL, BD Bioscience, USA). Subsequently, the membranes were incubated with anti-rabbit IgG secondary antibody conjugated to horseradish peroxidase (HRP) (1:1000 μL Abcam, Cambridge, UK). β-actin was simultaneously detected as a reaction control, by the anti-IgG monoclonal mouse antibody; 1:2000 (Abcam, Cambridge, MA, USA).

The HRP reaction products were visualized on Hyperfilme photographic film (Amersham, Little Chalfont, UK) after application of the ECL chemiluminescent kit (Bio-Rad, Hercules, USA). Quantitative densitometry of protein levels was performed on the J image. The bands obtained from the western blot were cut to assemble the figure in the results. The protein expression levels obtained were calculated and shown as mean ± S.E.M. of the average optical density and submitted to the t testt-test, with *p* < 0.05 considered a significant difference.

### 4.14. ELISA

The supernatant of the cells of the different experimental groups was collected during the other tests carried out. The protocol of the manufacturer BD Biosciences was followed for each cytokine/chemokine cytokines/chemokines analyzed, being IL-1β, IL-8 and MCP-1. Afterwards, the analysis was performed in a spectrophotometer at a wavelength of 450 nm. Data were analyzed by the *t*-test.

### 4.15. Immunocytochemistry

Cells (1 × 105) were grown on culture slides (Nunc, Naperville, IL, USA) and fixed in 4% paraformaldehyde, permeabilized in Triton X, washed with PBS-T, and subjected to blocking of non-specific binding in BSA (1%) and normal goat serum (3%). Cells were immunostained with mouse primary monoclonal antibodies (Ab) anti-p38/MAPK (BD Bioscience, USA) and anti-ERKpan (BD Biosciences, USA) diluted 1:200 followed by overnight incubation at 4 °C. After repeated washes in PBST (1%), the goat anti-mouse IgG antibody (Fc fragment specific, Dako, Glostrup, Denmark) conjugated to Alexa fluor 546 at a ratio of 1:200 (Molecular Probes, Eugene, Oregon, USA) was added, followed by 1 h incubation at room temperature. Finally, the cells were washed and the slides were mounted with DAPI mounting medium (Sigma Aldrich, Saint Louis, MO, USA). The analysis was performed using an Axioskop 2 fluorescence microscope (Zeiss, GR) equipped with a digital camera. Ten digital images of each replica were captured using the AxioVision software (Zeiss, GR) where six cells of each image were analyzed by densitometry obtained in the J image, which was statistically analyzed by the t test, with *p* < 0.05 considered a significant difference.

## 5. Conclusions

Our in vitro results demonstrated that piperine slows tumor progression in cervical cancer cells via reduced viability, cell proliferation, and colony formation, processes that are triggered by cell arrest in the cell cycle and apoptosis. These effects were mediated by piperine through the reduction in ERK, IL-1β, and IL-8 expression. Furthermore, piperine reduced cell migration by regulating gene and protein expression of MMPs and TIMPs, and regulating MCP-1 secretion.

Therefore, piperine reduces neoplastic evolution in vitro by acting on the PTGS2 pathway, which in turn regulates the secretion of cytokines and the expression of MMPs, MAPKs, and TIMPs. Piperine has proven to be a potential herbal medicine for the complementary treatment of cervical cancer; however, functional tests are necessary in the face of new technologies and aiming at clinical applications.

## Figures and Tables

**Table 1:** Piperine inhibitory concentration (IC_50_) in HeLa, SiHa, CaSki, and HaCaT cells at 24, 48, and 72 h.

	24 h	48 h	72 h
HeLa	208.0	151.0	69.9
SiHa	182.3	190.7	187.7
CaSki	167.8	83.3	104.2
HaCaT	218.4	162.5	181.7

**Table 2:** Primers used in real-time PCR.

Oligonucleotides	Sequency
*HPV16-E6* anti-sense	5′ CTACGTGTTCTTGATGATCTG 3′
*HPV16-E6* sense	5′ CTTACCACAGTTATGCACAGAG 3′
*HPV16-E7* anti-sense	5′ TGCCCATTAACAGGTCTTCC 3′
*HPV16-E7* sense	5′ ACAAGCAGAACCGGACAGAG 3′
*PTGS2* anti-sense	5′ AGAAGGCTTCCCAGCTTTTG 3′
*PTGS2* sense	5′ ATTCCCTTCCTTCGAAATGC 3′
*PTGER2* anti-sense	5′ AGGTCCCATTTTTCCTTTCG 3′
*PTGER2* sense	5′ CCACCTCATTCTCCTGGCTA 3′
*PTGER3* anti-sense	5′ TCTCCGTGTGTGTCTTGCAG 3′
*PTGER3* sense	5′ AGCTTATGGGGATCATGTGC 3′
*PTGER4* anti-sense	5′ CCAAACTTGGCTGATATAACTGG 3′
*PTGER4* sense	5′ CGAGATCCAGATGGTCATCTTAC 3′
*MMP2* anti-sense	5′ CCGTCAAAGGGGTATCCATC 3′
*MMP2* sense	5′ AAGTCTGGAGCGATGTGACC 3′
*MMP9* anti-sense	5′ ATTTCGACTCTCCACGCATC 3′
*MMP9* sense	5′ TTGTGCTCTTCCCTGGAGAC 3′
*TIMP1* anti-sense	5′ TTTTCAGAGCCTTGGAGGAG 3′
*TIMP1* sense	5′ ACTGTTGGCTGTGAGGAATG 3′
*TIMP2* anti-sense	5′ CTATATCCTTCTCAGGCCCTTTG 3′
*TIMP2* sense	5′ AGAAGGAAGTGGACTCTGGAAAC 3
*GAPDH anti-sense*	5′-ACCCACTCCTCCACCTTTGA-3
*GAPDH sente*	5′-CTGTTGCTGTAGCCAAATTCGT-3′

## Data Availability

Data is contained within the article.
